# TICAD9 and the UHC Knowledge Hub: Strengthening Japan-Africa Health Cooperation

**DOI:** 10.34172/ijhpm.9750

**Published:** 2026-07-07

**Authors:** Aminu Kende Abubakar, Usman Hafsat, Olukunmi Omobolanle Balogun, Shuhei Nomura

**Affiliations:** ^1^Kebbi State Ministry of Health, Birnin Kebbi, Nigeria.; ^2^Graduate School of Public Health, St. Luke’s International University, Tokyo, Japan.; ^3^Graduate School of Medicine, Juntendo University, Tokyo, Japan.; ^4^Department of Health Policy, National Center for Child Health and Development, Tokyo, Japan.; ^5^Global Health Policy Lab, International Research Institute of Disaster Science (IRIDeS), Tohoku University, Miyagi, Japan.; ^6^Keio University Global Research Institute (KGRI), Tokyo, Japan.; ^7^Department of Food, Nutrition and Health, Graduate School of Medicine, International University of Health and Welfare, Tokyo, Japan.

## Introduction

 The global health architecture is operating under deep uncertainty. Official development assistance for health is declining, donor priorities are shifting, and global health initiatives face growing pressure to demonstrate country alignment and institutional coherence.^1^ For African countries, these pressures compound long-standing constraints in health financing, workforce capacity, access to technology, and accountability, limiting progress toward universal health coverage (UHC).^2^ The Tokyo International Conference on African Development (TICAD), Japan’s main development framework for partnership with Africa since 1993, provides an important platform for responding to these challenges.^3^ The Ninth Tokyo International Conference on African Development (TICAD9), held in Yokohama in August 2025, came at a time when Africa’s health priorities require more coherent and accountable forms of international cooperation.^4^ At the December 2025 UHC High-Level Forum in Tokyo, the UHC Knowledge Hub was formally launched as a joint initiative involving Japan’s Ministry of Finance, Japan’s Ministry of Health, Labour and Welfare, the World Bank, and the World Health Organization (WHO).^5^ This Viewpoint argues that TICAD9 gives Japan an important opportunity to strengthen its health partnership with Africa. Japan’s most useful contribution is institutional support: helping African governments strengthen health financing, reduce fragmentation, and hold partners accountable within African-led frameworks. The UHC Knowledge Hub is central to this opportunity because it brings health and finance actors into the same policy space. However, Japan must respond to Africa’s structural constraints while also recognizing the policy innovations, institutions, and priorities that African countries bring to the partnership.

## Africa’s Structural Health-System Constraints

 A first constraint is fragmentation in African health systems, which is often framed as a coordination problem that can be solved through better planning tools or donor dialogue. The evidence suggests a deeper problem. In Malawi, for example, 55% of total health expenditure in 2020 flowed through 166 external financing sources and 265 implementing partners, while external dependence reached 80% for HIV programming; government health expenditure remained at US $9.60 per capita, far below the estimated minimum needed to deliver essential services.^6^ Across many African countries, external financing remains organized around separate donor priorities, disease-specific funding streams, and parallel reporting requirements. These arrangements impose high transaction costs on governments and undermine strategic planning. They also persist because they reflect the incentives of the current global health architecture: donors seek visible and attributable results, implementing agencies compete for disease-specific resources, and recipient governments are left to align multiple partners whose timelines and reporting systems do not always match national plans.^7^ The Lusaka Agenda recognized these problems and called for strategic shifts in global health initiatives, including stronger contributions to primary healthcare, better alignment with national systems, catalytic domestic financing, and greater operational coherence.^8^ Fragmentation is reinforced by limited fiscal space. More than two decades after the Abuja Declaration, over 30 African Union (AU) member states still allocate less than 10% of national budgets to health, below the 15% benchmark.^2,9^ Debt pressures further reduce room for health investment; in several countries, external debt service now rivals or exceeds public health expenditure.^10^ Calls for greater domestic resource mobilization are therefore unlikely to succeed without broader reforms in debt, taxation, concessional financing, and partner alignment. Mechanisms designed to catalyze domestic investment may also fall short when they rely heavily on loans or when external financing substitutes for, rather than adds to, public spending.^11^ These financing constraints interact with other structural pressures, including health workforce shortages, weak data systems for planning and accountability, limited access to technology transfer, and continued dependence on external partners for priority-setting and implementation support.^12,13^ For Japan, the opportunity is to support African governments in addressing these linked constraints through more coherent financing, stronger institutional capacity, and closer alignment with African-led frameworks.

## TICAD9 in the Evolution of Japan-Africa Health Cooperation

 TICAD has evolved since its launch in 1993 from a Japan-led development forum into a broader platform for dialogue on African priorities, co-hosted with the United Nations, the United Nations Development Program, the World Bank, and the AU Commission.^3^ TICAD8, held in Tunis in 2022, pledged US $30 billion over three years across public and private contributions, including health, human resources, development finance, green growth, regional stability, and food security.^3^ However, limited independent tracking across TICAD cycles makes it difficult to assess how far these commitments translated into sustained health-system outcomes. TICAD9, held in Yokohama from 20 to August 22, 2025, came at a time when African health systems faced increasing pressure from declining aid, fiscal constraints, and demands for stronger country ownership. TICAD9’s mechanisms, outlined in the Yokohama Declaration, seek to address these constraints.^4^ The One Stop Shop model aims to align support within country frameworks, while the UHC Knowledge Hub focuses on institutional capacity in health financing, governance, and policy design.^14^ This signals an evolution from project-based assistance. However, the effectiveness of both initiatives depends critically on their integration with existing African-led frameworks, notably the Africa Centres for Disease Control and Prevention’s (Africa CDC’s) strategic priorities and the Lusaka Agenda.^8,12^ Such integration is essential to prevent the creation of parallel structures that deepen, rather than resolve, fragmentation. The Yokohama Declaration’s commitments to digital transformation, workforce development, and local manufacturing (through initiatives such as the Platform for Harmonized African Health Manufacturing, the Africa Medicines Agency, and the AU’s digital transformation strategy) are well-aligned with Africa’s stated priorities.^12^ Their value, however, will depend on policy coherence. Workforce development should not be undermined by labor-mobility agreements that encourage emigration.^15^ Local manufacturing requires genuine technology transfer, not only equipment provision, while digital transformation requires infrastructure investments and data governance frameworks that extend beyond equipment provision.^16^ TICAD9 can strengthen Japan-Africa health cooperation if these commitments are translated into sustained support for African-led priorities, with clear follow-up and measurable accountability.

## The UHC Knowledge Hub in Japan-Africa Health Cooperation

 The UHC Knowledge Hub is a joint initiative involving Japan’s Ministry of Finance, Japan’s Ministry of Health, Labour and Welfare, the World Bank Group, and the WHO. It was launched to support low- and middle-income countries in strengthening sustainable financing for UHC, with a focus on senior officials from ministries of health and finance.^5^ Its importance for Japan-Africa health cooperation lies in this finance-health interface. This is where decisions about fiscal space, budget rules, domestic resource mobilization, and alignment with external financing are made. The interface is therefore central to addressing the fiscal pressures and financing fragmentation that constrain African health systems. The Hub is expected to combine advocacy, training programs, and implementation support, while also supporting country-led initiatives such as National Health Compacts.^5^ These functions speak directly to the problems identified in the Lusaka Agenda, including weak alignment between global health initiatives and national systems, limited support for primary healthcare, and the need to catalyze sustainable domestic financing.^8,11^ Its connection to the WHO Council on the Economics of Health for All is also important because it frames health as an investment in social and economic development, not only as a budgetary cost.^17^ For African countries facing debt pressure and limited fiscal space, this framing could help move UHC discussions beyond health-sector allocation alone toward broader questions of fiscal policy, debt, and public investment. The Hub’s global mandate means African priorities will not be automatically reflected. African governments and continental institutions need to shape their agenda from the start. This is feasible because five of the eight inaugural cohort countries are African: Egypt, Ethiopia, Ghana, Kenya, and Nigeria.^5^ Their participation places African financing realities near the center of the Hub’s early learning. These countries differ in income and health-system context, but share fiscal pressures, including constrained public spending, debt-service burdens, and the need to align external financing with domestic reforms. The wider test is whether lessons from these countries are translated into tools that can support non-participating countries through the Africa CDC, the WHO Regional Office for Africa, and other African-led platforms. The Forum is expected to be convened regularly in Tokyo to review progress on the Hub and improve its activities.^5^ Its work should also connect to the 2027 United Nations High-Level Meeting on UHC, where progress on UHC commitments will be reviewed.

## Co-Creation as the Basis of TICAD9 Health Partnership

 TICAD9 framed Japan-Africa cooperation around the goal of “co-creating innovative solutions with Africa,” grounded in African ownership, international partnership, openness, and mutual interest.^4^ This framing should guide the health partnership. Japan brings valuable experience in UHC, finance-health coordination, technology, and aging-related care. African countries also bring important experience in community-based primary healthcare, frontline service delivery in underserved areas, digital adaptation, regional health governance, and health-system resilience. Co-creation means combining strengths rather than viewing Japan as the sole source of knowledge and Africa as a passive recipient. The TICAD9 thematic event on the *Africa-Japan Common Vision on Health: Co-creating Health Security and Sustainable Growth* translated this principle into five priorities: sustainable health financing, diagnostics and health data systems, integrated and digitalized primary and preventive care, co-led research and technology innovation, and pandemic preparedness and health-system resilience.^18^ These priorities offer a practical agenda for linking Japan’s institutional and technological strengths with African-led priorities in financing, community systems, local production, digital transformation, and health security.

## Conditions for Structural Change and Accountability Metrics

 The value of TICAD9 and the UHC Knowledge Hub will depend less on the ambition of their declarations than on whether they help address the structural conditions that weaken African health systems. The first condition, expanded fiscal space, calls for greater domestic health investment, which remains difficult to realize unless it is linked to debt relief or restructuring where needed, fairer tax arrangements, improved concessional financing, and stronger alignment of external resources with national budgets and plans.^2,8,11^ The second is genuine technology transfer: TICAD9’s commitments to local production and support for the Africa Medicines Agency will matter only if they help build African capacity in manufacturing, regulation, and procurement, rather than merely provide equipment or short-term technical support. This requires attention to intellectual property, market shaping, pooled procurement, quality assurance, and long-term industrial partnerships.^12^ The third is policy coherence, support for African health systems should be assessed across sectors, not only within health aid.^8,11^ Workforce development, for example, should not be undermined by labor-mobility arrangements that accelerate health worker depletion without safeguards for source countries. Digital transformation should include investment in infrastructure, interoperability, data governance, and local capacity, not only the introduction of new tools. Accountability should therefore focus on a small set of measurable signals across short-, medium-, and long-term horizons. By the 2027 United Nations High-Level Meeting on UHC, Hub-supported African countries should report whether National Health Compacts are linked to medium-term expenditure frameworks and domestic financing plans. By the next TICAD, Japan-supported health partnerships, including private-sector collaborations, should demonstrate durable capacity building, such as technology transfer, regulatory strengthening, or institutional training embedded within African systems. Progress should be reported through African-led accountability mechanisms, including Africa CDC and Lusaka Agenda follow-up processes, with inputs from the UHC Knowledge Hub, Japan, WHO, the World Bank, and participating African governments.

## Conclusion

 At a time when the wider aid architecture is becoming less reliable, Japan-Africa health cooperation has renewed importance. TICAD9 is distinctive because it builds on long-standing political trust and multilateral legitimacy, while the UHC Knowledge Hub gives the partnership a timely focus on health financing as African countries advance stronger domestic financing and country-led health systems on the path to 2030. In this context, co-creation matters because it moves cooperation beyond donor-recipient relations toward a partnership shaped by mutual interest and African ownership. Progress should be reviewed against time-bound signals linked to financing plans, durable capacity building, and African-led accountability mechanisms. Africa’s demand is clear: partnerships that expand fiscal space, enable access to technology, and, more importantly, remain accountable to African-led priorities. The test for TICAD9 and the Hub is whether they can help make this form of cooperation routine.

## Disclosure of artificial intelligence (AI) use

 During preparation, the authors used Gemini AI to improve grammar, revise, and shorten the manuscript to meet the journal’s word count. They reviewed, edited, and took full responsibility for the published article.

## Ethical issues

 Not applicable.

## Conflicts of interest

 Authors declare that they have no conflicts of interest.

## Disclaimer

 The opinions expressed are solely those of the authors and do not necessarily reflect the views of their organization.
